# Associations between blood-based biomarkers of Alzheimer’s disease with cognition in motoric cognitive risk syndrome: A pilot study using plasma Aβ42 and total tau

**DOI:** 10.3389/fnagi.2022.981632

**Published:** 2022-10-04

**Authors:** Pei-Hao Chen, Sang-I Lin, Ying-Yi Liao, Wei-Ling Hsu, Fang-Yu Cheng

**Affiliations:** ^1^Department of Neurology, MacKay Memorial Hospital, Taipei, Taiwan; ^2^Department of Medicine, MacKay Medical College, New Taipei City, Taiwan; ^3^Graduate Institute of Mechanical and Electrical Engineering, National Taipei University of Technology, Taipei, Taiwan; ^4^Institute of Long-Term Care, MacKay Medical College, New Taipei City, Taiwan; ^5^Department of Gerontological Health Care, National Taipei University of Nursing and Health Sciences, Taipei, Taiwan; ^6^Center of Dementia Care, MacKay Memorial Hospital, New Taipei City, Taiwan

**Keywords:** plasma biomarker, cognition, gait speed, Alzheimer’s disease, motoric cognitive risk syndrome

## Abstract

**Background:**

Motoric cognitive risk (MCR) syndrome is a conceptual construct that combines slow gait speed with subjective cognitive complaints and has been shown to be associated with an increased risk of developing dementia. However, the relationships between the pathology of Alzheimer’s disease (AD) and MCR syndrome remain uncertain. Therefore, the purpose of this study was to determine the levels of plasma AD biomarkers (Aβ42 and total tau) and their relationships with cognition in individuals with MCR.

**Materials and methods:**

This was a cross-sectional pilot study that enrolled 25 individuals with normal cognition (NC), 27 with MCR, and 16 with AD. Plasma Aβ42 and total tau (t-tau) levels were measured using immunomagnetic reduction (IMR) assays. A comprehensive neuropsychological assessment was also performed.

**Results:**

The levels of plasma t-tau proteins did not differ significantly between the MCR and AD groups, but that of plasma t-tau was significantly increased in the MCR and AD groups, compared to the NC group. Visuospatial performance was significantly lower in the MCR group than in the NC group. The levels of plasma t-tau correlated significantly with the Montreal Cognitive Assessment (MoCA) and Boston naming test scores in the MCR group.

**Conclusion:**

In this pilot study, we found significantly increased plasma t-tau proteins in the MCR and AD groups, compared with the NC group. The plasma t-tau levels were also significantly correlated with the cognitive function of older adults with MCR. These results implied that MCR and AD may share similar pathology. However, these findings need further confirmation in longitudinal studies.

## Introduction

Motoric cognitive risk (MCR) syndrome is a conceptual construct that combines slow gait speed with subjective cognitive complaints (Verghese et al., 2013). Motoric cognitive risk syndrome is easy to screen in the community and is associated with an increased risk of developing dementia, such as Alzheimer’s disease (AD) or vascular dementia (Verghese et al., 2013, 2014a). This syndrome is presented in approximately 10% of the older population and has been shown to be associated with an increased risk of falls and disability (Verghese et al., 2014b). The pathophysiological mechanisms underlying MCR have not yet been fully established. Previous studies revealed that MCR could be related to cortical atrophy (Blumen et al., 2021), lacunar infarction (Wang et al., 2016), or increased levels of systematic inflammatory biomarkers (Bortone et al., 2021). A cross-sectional study using MRI found that gray matter volumes, hippocampal volumes, and white matter hyperintensities were worse in individuals with MCR (Yaqub et al., 2022). Meiner et al. (2021) also reported that Apolipoprotein E ε4 allele (APOE ε4) was associated with the conversion to dementia in older adults with MCR. On the contrary, although MCR has good predictive validity for the incidence of AD, the relationships between AD pathology and MCR remain uncertain. Alzheimer’s disease is characterized by two major pathological lesions in the brain, amyloid β plaques and tau neurofibrillary tangles (Avila, 2006; Capetillo-Zarate et al., 2012). Alzheimer’s disease pathology has been found to have a strong association with gait disorder in older adults without dementia and can predict cognitive decline and incident dementia (Del Campo et al., 2016; Tian et al., 2017). Recently, in a study investigating the relationships between MCR and AD pathology using imaging biomarkers, no significant differences in the biomarkers of AD, such as amyloid, tau deposition, or white matter hyperintensities, were found between individuals with MCR and without MCR (Bommarito et al., 2022). Although the findings of this study were informative, the sample size was relatively small, and no comparisons were made with the normal cognition (NC) group, making it difficult to determine whether the physiological changes in MCR were caused by normal aging or the pathological changes related to AD.

Amyloid β and tau protein are presented in the cerebrospinal fluid (CSF) and the brain and can be measured by lumbar puncture (Olsson et al., 2016) or positron emission tomography (PET), respectively (Johnson et al., 2013). However, their clinical applications are often limited because CSF sampling is invasive and PET scans are costly. Recently, blood-based biomarkers have been used cost-effectively to assist in the diagnosis of neurodegenerative diseases and the estimation of prognosis clinically (Hansson, 2021). Immunomagnetic reduction (IMR) is a highly sensitive assay technology for the analysis of biomarkers in blood samples, and can accurately quantify plasma Aβ and tau (Baird et al., 2015; Yang et al., 2017). A review article including 15 studies using the IMR assay indicated that significant increases in the plasma levels of Aβ42 and tau in persons with amnestic mild cognitive impairment (aMCI) and AD, and the levels of Aβ42 and tau are related to the severity of AD (Lee et al., 2022). These findings suggested that assays of plasma Aβ42 or tau using IMR may be promising tools for facilitating an early diagnosis of AD. Therefore, measuring plasma Aβ42 or tau protein concentrations *via* IMR assay in individuals with MCR may also reveal its associated physiological changes.

In this pilot study, we compared the levels of plasma Aβ42 and total tau (t-tau), and their associations with global and specific cognitive functions among older adults with NC, individuals with MCR, and those with AD. We aimed to determine the levels of plasma AD biomarkers (Aβ42 and total tau) and their relationships with cognition in individuals with MCR.

## Materials and methods

### Participants and study design

This work was a cross-sectional study conducted in Taiwan between June 2021 and April 2022. Participants were recruited from a medical center in Taipei. All participants met the following criteria: (a) aged 60 years or older, (b) could walk 10 m independently, and (c) living in the community. The exclusion criteria were as follows: (a) unstable medical conditions, for example, the presence of major visual or hearing loss, and (b) a recent or planned surgery leading to limitations in walking or participation in this study. A total of 68 participants provided informed consent, and the study procedures were approved by the Institutional Review Board of Mackay Memorial Hospital (number: 21MMHIS078e). We confirmed that the experimental procedures were performed in accordance with the relevant guidelines and regulations. Of these participants, 16 suffered from mild to moderate AD, with the disease courses longer than 2 years. There were 27 participants with MCR, with the symptoms courses longer than 3 months. There were 25 NC controls. Clinical diagnosis was based on physical and neurological examinations, laboratory tests, and comprehensive neuropsychological tests.

### Demographic and clinical measures

Information about age, sex, history of metabolic disease, and level of education was obtained from interviews and medical charts. All the participants completed the comprehensive neuropsychological testing, including the Mini-Mental State Examination (MMSE), Montreal Cognitive Assessment (MoCA), clinical dementia rating (CDR), the Chinese version of Parts A and B of the trail making test (TMT) (Wu et al., 2015), category fluency test (Rosen, 1980), digital symbol modality test, the Chinese version of the California Verbal Learning Test (CVLT-SF) (Chang et al., 2010), Judgment of Line Orientation test (Mitrushina et al., 2005), Boston naming test (del Toro et al., 2011), and the Chinese version of the Geriatric Depression Scale-15 (GDS-15) (Liu et al., 1997) to evaluate global cognitive function, executive function, attention and working memory, episodic memory, visuospatial performance, language, and depression. All the neuropsychological tests were assessed by experienced neurologists who have received professional neuropsychological scale training.

### Motoric cognitive risk syndrome group

This study mainly adopted the diagnostic criteria of Verghese et al. (2014b). The inclusion criteria for the MCR group were as follows: (1) subjective cognitive complaints, (2) slow gait speed, (3) absence of dementia, and (4) a consistent level of independence in the activities of daily living. The presence of subjective cognitive complaints was determined by a “yes” response to the memory item on the Geriatric Depression Scale (Yesavage et al., 1982; Ayers and Verghese, 2019). Slow gait speed was defined as a walking speed of one or more standard deviations (SDs) below the mean age- and sex-specific gait speed (Verghese et al., 2013, 2014b). The exclusion criteria were (1) consumption of any medications causing cognitive complaints during the past 3 months, (2) inability to cooperate with the procedures and tests in this study, and (3) other significant neurological or psychiatric conditions, such as Parkinson’s disease, stroke, Parkinsonism, other movement disorder, or major depression.

### Alzheimer’s disease group

The presence of AD was based on the diagnostic criteria defined by the National Institute on Aging (NIA) and the Alzheimer’s Association (AA) in McKhann et al. (2011). The inclusion criteria were as follows: (1) fulfilled the core clinical criteria proposed by the NIA-AA workgroup, and (2) clinical dementia rating (CDR) = 1 (Morris, 1997). The exclusion criteria were as follows: (1) other significant neurological or psychiatric conditions that might cause cognitive impairments, such as frontotemporal dementia, dementia with Lewy bodies, vascular dementia, Parkinson’s disease, stroke, vitamin B12 deficiency, alcoholism, or major depression.

### Normal cognition group

Older adults with NC were screened and recruited from the neurology outpatient department. The inclusion criteria were as follows: (1) no subjective or objective memory deficits or declines in cognitive performance, (2) no mental or neurological disease, and (3) CDR = 0 (Morris, 1997). The exclusion criteria were the same as those for the MCR group.

### Blood sample processing and immunomagnetic reduction assays

Ten milliliters of venous blood were collected from each participant, and then the blood sample was centrifuged at room temperature for 15 min at 1,500–2,500 xg. Pure serum was drained and immediately frozen in test tubes at −80°C. Frozen plasma was dry ice delivered to MagQu Co., Ltd. (New Taipei City, Taiwan) for IMR assay processing. The technical information and the validation accuracy of the IMR assay were previously described (Chiu et al., 2013; Lue et al., 2017). The volumes of the t-tau magnetic reagents and the to-be-detected samples were 80 and 40 μl, respectively. The volumes of the Aβ42 magnetic reagents and the to-be-detected samples were 60 and 60 μl, respectively. After mixing, superconducting quantum interference device (SQID)-based alternative current magnetosusceptometer (model XacPro-S, MagQu Co., New Taipei City, Taiwan) to determine the time-dependent alternative current magnetic susceptibility. Because of the association between the antibody-functionalized magnetic nanoparticles and the target biomarkers, the alternative current magnetic susceptibility of the mixture was reduced. This reduction in magnetic susceptibility is referred to as the IMR signal. For each to-be-detected sample, the sample was divided into three parts, and the IMR signals of each part were detected individually. Therefore, three IMR signals were obtained for each sample. The mean value, standard deviation, and coefficient of variation (interrun) of the IMR signals were analyzed.

### Statistical analysis

Analysis was conducted using Statistical Product and Service Solutions (SPSS) version 26.0 (SPSS Inc., Chicago, IL, USA). The participants’ characteristics are summarized using the means, SDs, or numbers, as appropriate. Between-group comparisons were performed using one-way analysis of covariance (ANCOVA) (continuous variables) (age and sex as covariate variables), followed by Bonferroni *post-hoc* tests, or chi-square tests (categorical variables). Spearman’s rank correlation coefficient was used to examine the correlations between plasma neurodegenerative biomarkers and cognitive functions in the NC, MCR, and AD groups. In this study, the within-group Spearman’s rank correlations were examined 10 times. Although such multiple tests might inflate the type I error, previous studies have shown that correlation coefficients larger than 0.3 could indicate meaningful correlations without correction for multiple comparisons (Hinkle et al., 2003; Mukaka, 2012). Thus, for the correlational analysis, a correlation coefficient greater than 0.3 or a *p*-value less than 0.05 would be considered significant.

## Results

### Baseline demographics and biomarkers

Sixty-eight participants were recruited for the study. Table 1 presents the characteristics and plasma biomarkers of the participants in the NC (*N* = 25), MCR (*n* = 27), and AD (*n* = 16) groups. The mean ages of the participants in the NC, MCR, and AD groups were 74.3 ± 7.4, 75.2 ± 6.4, and 78.2 ± 6.6 years, respectively. There were no differences in age, body mass index, educational level, or the prevalence of medical conditions (hypertension, diabetes, and heart disease) among the three groups. However, the AD group had a higher proportion of women than the other groups (NC: 36%, MCR: 52%, AD: 81%, *p* = 0.018). The MCR and AD groups had poorer general cognitive functions (MMSE, *F* = 25.740, η^2^ = 0.450, *p* < 0.001; MoCA, *F* = 11.911, η^2^ = 0.274, *p* < 0.001; CDR, *F* = 156.595, η^2^ = 0.833, *p* < 0.001) and lower gait speed (*F* = 29.422, η^2^ = 0.487, *p* < 0.001) than the NC group. The AD group had significantly lower MMSE (Cohen’s *d* = 1.675, *p* < 0.001), MoCA (Cohen’s *d* = 1.058, *p* = 0.014), and CDR (Cohen’s *d* = 3.299, *p* < 0.001) scores but a faster gait speed (Cohen’s *d* = 0.784, *p* = 0.032) than the MCR group. Compared to the NC group, the AD group had higher levels of plasma t-tau (Cohen’s *d* = 1.279, *p* < 0.001). The levels of plasma Aβ42 did not differ significantly between the MCR and NC groups, but the former had significantly higher levels of t-tau (Cohen’s *d* = 0.781, *p* = 0.018).

**TABLE 1:** Comparison of participants’ characteristics among the three groups (*n* = 68).

	Normal cognition (*n* = 25)	Motoric cognitive risk syndrome (*n* = 27)	Alzheimer’s disease (*n* = 16)	*F*	*P*-value	Partial eta squared
Age (years)	74.3 ± 7.4	75.2 ± 6.4	78.2 ± 6.6		0.204	
Sex (male/female), n	16/9	13/14	3/13		0.018	
BMI	24.9 ± 2.6	24.2 ± 3.4	23.9 ± 3.9		0.586	
Education level	7.8 ± 4.7	6.4 ± 4.9	5.2 ± 4.8		0.489	
0 years, n	3	5	4			
1–6 years, n	12	17	7			
7–12 years, n	7	2	4			
13 or more years, n	3	3	1			
Hypertension, n	10	17	7		0.216	
Diabetes mellitus, n	6	13	5		0.177	
Heart disease, n	3	6	2		0.546	
MMSE	26.6 ± 2.8	23.7 ± 4.6	15.3 ± 5.4**^##^	25.745	<0.001^a^	0.450
MoCA	22.2 ± 4.5	17.9 ± 6.2*	11.6 ± 5.7**^#^	11.911	<0.001^a^	0.274
CDR	0.0 ± 0.0	0.3 ± 0.3**	1.0 ± 0.0**^##^	156.595	<0.001^a^	0.833
Gait speed, m/s	1.1 ± 0.2	0.6 ± 0.2**	0.8 ± 0.3*^#^	29.422	<0.001^a^	0.487
Aβ42, pg/ml	16.4 ± 0.5	16.6 ± 0.6	16.8 ± 0.5	2.328	0.106^a^	0.069
t-tau, pg/ml	20.6 ± 1.7	22.7 ± 3.4*	24.2 ± 3.6**	9.583	<0.001^a^	0.233

BMI, body mass index; MMSE, Mini-Mental State Examination; MoCA, Montreal Cognitive Assessment; CDR, clinical dementia rating; Aβ42, amyloid beta 42; t-tau, total tau.

^a^Adjusted for age and sex.

* and **: Significance levels of <0.05 and <0.001 for intergroup comparisons between the normal cognition (NC) group and other groups.

^#^ and ^##^: Significance levels of <0.05 and <0.001 for intergroup comparisons between the motoric cognitive risk (MCR) syndrome group and the Alzheimer’s disease (AD) group.

### Cognitive function

The cognitive function of the participants is shown in Table 2. The MCR and AD groups had poorer visuospatial performance (Judgment of Line Orientation, *F* = 7.591, η^2^ = 0.194, *p* = 0.001) than the NC group. The AD group had significantly poorer executive function (TMT-A, Cohen’s *d* = 1.265, *p* = 0.013), attention and working memory (Digital symbol modality test, Cohen’s *d* = 1.318, *p* = 0.025), episodic memory (CVLT-SF, Cohen’s *d* = 0.945, *p* = 0.015), and language ability (Boston naming test, Cohen’s *d* = 1.831, *p* < 0.001) than the NC group. The AD group had significantly poorer language performance (Boston naming test, Cohen’s *d* = 1.523, *p* < 0.001) than the MCR group.

**TABLE 2:** Comparison of participants’ cognitive performance and physical function among the three groups (*n* = 68).

	Normal cognition (*n* = 25)	Motoric cognitive risk syndrome (*n* = 27)	Alzheimer’s disease (*n* = 16)	*F*	*P*-value	Partial eta squared
**Executive function**						
TMT-A, s	23.1 ± 15.1	42.6 ± 32.1	61.5 ± 40.2*	4.679	0.013^a^	0.129
TMT-B, s	62.0 ± 36.0	83.6 ± 35.5	89.9 ± 37.2	1.892	0.159^a^	0.057
Category fluency test	11.3 ± 3.6	10.0 ± 3.8	7.8 ± 4.4	2.060	0.136^a^	0.061
**Attention and working memory**						
Digital symbol modality test	34.0 ± 13.3	23.7 ± 15.6	15.1 ± 15.3*	4.284	0.018^a^	0.120
**Episodic memory**						
CVLT-SF	18.8 ± 5.3	15.9 ± 5.3	13.3 ± 6.3*	4.393	0.016^a^	0.122
**Visuospatial performance**						
Judgment of line orientation	13.6 ± 3.6	9.4 ± 4.3*	7.3 ± 5.1*	7.591	0.001^a^	0.194
**Language**						
Boston naming test	23.5 ± 5.1	21.3 ± 4.5	12.7 ± 6.6**^##^	15.175	<0.001^a^	0.325
**Depression**						
Geriatric Depression Scale-15	3.1 ± 2.8	3.8 ± 3.2	2.6 ± 2.7	0.698	0.501^a^	0.022

TMT, trail making test; CVLT-SF, California Verbal Language Test-Short Form.

^a^Adjusted for age and sex.

* and **: Significance levels of <0.05 and <0.001 for intergroup comparisons between the normal cognition (NC) group and other groups.

^##^: Significance level of <0.001 for intergroup comparisons between the motoric cognitive risk (MCR) syndrome group and the Alzheimer’s disease (AD) group.

### Association of cognitive function and biomarkers in motoric cognitive risk “Syndrome”

**FIGURE 1:**
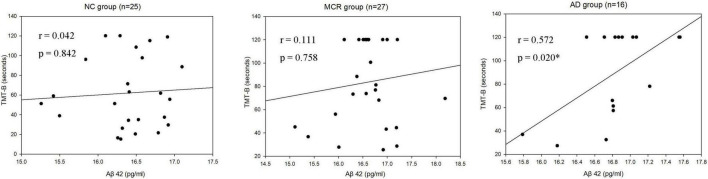
Scatterplots of the associations between TMT-B and plasma Aβ42 levels (pg/ml) in the three groups. NC, normal cognition; MCR, Motoric Cognitive Risk syndrome; AD, Alzheimer’s disease; Aβ42, amyloid beta 42; TMT-B, trail making test-part B; *p < 0.05.

**FIGURE 2:**
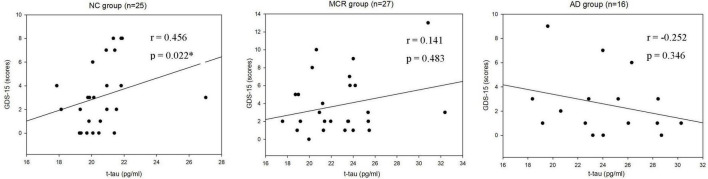
Scatterplots of the associations between Geriatric Depression Scale-15 (GDS-15) scores and plasma t-tau levels (pg/ml) in the three groups. NC, normal cognition; MCR, Motoric Cognitive Risk syndrome; AD, Alzheimer’s disease; t-tau, total tau; GDS-15, Geriatric Depression Scale-15; *p < 0.05.

**FIGURE 3:**
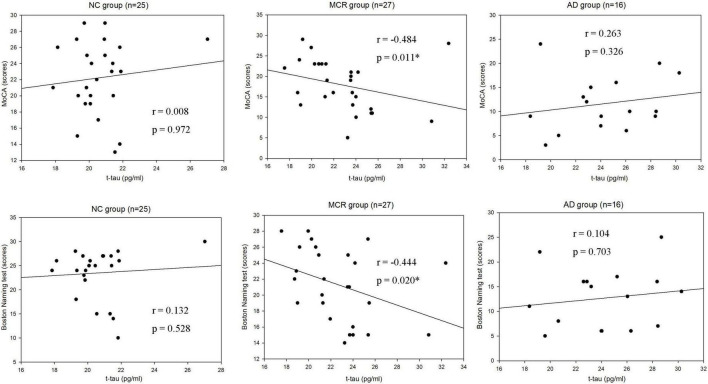
Scatterplots of the associations between Montreal Cognitive Assessment (MoCA) scores, Boston Naming test scores, and plasma t-tau levels (pg/ml) in the three group. NC, normal cognition; MCR, Motoric Cognitive Risk syndrome; AD, Alzheimer’s disease; t-tau, total tau; MoCA, Montreal Cognitive Assessment; *p < 0.05.

Table 3 shows the correlations between cognitive function and plasma Aβ42 in the NC, MCR, and AD groups. In the AD group, the levels of plasma Aβ42 were positively correlated with the TMT-B scores (*r*_s_ = 0.572, *p* = 0.020), which indicated that higher plasma Aβ42 levels were associated with lower cognitive function (Figure 1). However, there were no significant correlations between the levels of plasma Aβ42 and cognitive function in the NC and MCR groups. Table 4 shows the correlations between cognitive function and plasma t-tau in the NC, MCR, and AD groups. In the NC group, the levels of plasma t-tau were positively correlated with the GDS-15 scores (*r*_s_ = 0.456, *p* = 0.022), which indicated that higher plasma t-tau levels were associated with higher levels of depression (Figure 2). In the MCR group, the levels of plasma t-tau were negatively correlated with the MoCA and Boston naming test scores [MoCA, *r*_s_ = (−0.484), *p* = 0.011; Boston naming test, *r*_s_ = (−0.444), *p* = 0.020], which indicated that higher plasma t-tau levels were associated with lower cognitive function (Figure 3). However, there were no significant correlations between the levels of plasma t-tau and cognitive function in the AD group.

**TABLE 3:** Associations between plasma Aβ42 and cognitive function in the three groups.

	Normal cognition (*n* = 25)		Motoric cognitive risk syndrome (*n* = 27)		Alzheimer’s disease (*n* = 16)	
Outcomes	*r*_s_	*p*	*r*_s_	*p*	*r*_s_	*P*
MMSE	0.232	0.264	−0.096	0.633	−0.221	0.412
MoCA	−0.151	0.472	0.034	0.865	−0.105	0.699
TMT-A	0.038	0.855	0.067	0.741	0.353	0.180
TMT-B	0.042	0.842	−0.011	0.958	0.572	0.020*
Category fluency test	−0.156	0.457	0.227	0.254	0.341	0.197
CVLT-SF	−0.020	0.926	−0.052	0.795	−0.246	0.359
Digital symbol modality test	−0.260	0.210	−0.068	0.737	−0.387	0.139
Judgment of line orientation	0.053	0.801	−0.258	0.193	−0.006	0.983
Boston naming test	−0.340	0.096	−0.091	0.653	−0.289	0.278
Geriatric Depression Scale-15	−0.017	0.935	0.145	0.471	0.408	0.116

Aβ42, amyloid beta 42; t-tau, total tau; MoCA, Montreal Cognitive Assessment; TMT, trail making test; CVLT-SF, California Verbal Language Test-Short Form; *p < 0.05.

**TABLE 4:** Associations between plasma t-tau and cognitive function in the three groups.

	Normal cognition (*n* = 25)		Motoric cognitive risk syndrome (*n* = 27)		Alzheimer’s disease (*n* = 16)	
Outcomes	*r*_s_	*p*	*r*_s_	*p*	*r*_s_	*p*
MMSE	−0.121	0.564	−0.359	0.066	0.006	0.983
MoCA	0.008	0.972	−0.484	0.011*	0.263	0.326
TMT-A	0.073	0.730	0.338	0.085	−0.287	0.280
TMT-B	0.001	0.999	0.194	0.332	−0.143	0.598
Category fluency test	−0.260	0.210	−0.061	0.764	0.163	0.545
CVLT-SF	0.055	0.793	−0.064	0.750	0.244	0.362
Digital symbol modality test	−0.163	0.436	−0.356	0.068	−0.074	0.786
Judgment of line orientation	−0.317	0.122	−0.358	0.067	0.091	0.736
Boston naming test	0.132	0.528	−0.444*	0.020	0.104	0.703
Geriatric Depression Scale-15	0.456	0.022*	0.141	0.483	−0.252	0.346

Aβ42, amyloid beta 42; t-tau, total tau; MoCA, Montreal Cognitive Assessment; TMT, trail making test; CVLT-SF, California Verbal Language Test-Short Form; *p < 0.05.

## Discussion

To our knowledge, this is the first study to use plasma biomarkers to examine Aβ42 and t-tau in relation to cognitive function in older adults with MCR and the first to report that plasma t-tau is related to cognitive function in individuals with MCR. We found that the levels of plasma Aβ42 and t-tau did not differ between older adults with MCR and AD, but the levels of plasma t-tau in older adults with MCR and AD were significantly higher than those in older adults with NC. In addition, the levels of plasma t-tau in individuals with MCR were related to MoCA and Boston naming test scores.

The accumulation of Aβ plaques and tau neurofibrillary tangles in the brain are two pathological hallmarks of AD (Avila, 2006; Capetillo-Zarate et al., 2012). These biomarkers can cause neural damage and lead to hippocampal atrophy, cortical shrinkage, and brain damage (Pini et al., 2016; Pereira et al., 2017). In the present study, the AD group had higher levels of plasma t-tau, compared with the NC group. These findings reconfirmed the above statement. MCR is a symptomatic predementia phase and has good predictive validity for the incidence of dementia (Verghese et al., 2013, 2014a). However, previous studies have presented inconsistent results regarding whether MCR would increase the risk of AD. In the Einstein Aging Study, MCR was a strong predictor of vascular dementia (hazard ratio = 12.81) but not AD dementia (hazard ratio = 0.66) (Verghese et al., 2013). In another multicountry study, samples from multiple cohorts were pooled, and it was found that MCR was associated with an increased risk of AD in two cohorts (hazard ratio = 1.79–2.10) (Verghese et al., 2014a). Therefore, clarifying the relationship between AD pathology and MCR may aid clinical diagnosis and the planning of prevention strategies. To date, only one study has investigated the relationship between MCR and AD pathology using imaging biomarkers (Bommarito et al., 2022) and reported findings inconsistent with the present study. In the above mentioned study, 20 patients with cognitive complaints (8 individuals with MCR and 12 without MCR) were recruited from the Memory Center of the Geneva University Hospitals, and no significant differences in the deposition of amyloid and tau protein in the brain between older adults with MCR and without MCR were found. The individuals without MCR in Bommarito et al. (2022) had subjective cognitive complaints and a CDR scale score of 0.5, which might have affected the results of the study. The strength of our study was that NC and AD groups were included for comparison. We found that the levels of plasma t-tau were higher in the participants with MCR and AD than in the older adults with NC. However, plasma t-tau did not differ between the individuals with MCR and those with AD. MCR is a predementia syndrome similar to mild cognitive impairment (MCI). A review of 15 clinical studies demonstrated an increase in the plasma t-tau levels in older adults with MCI and AD (Lee et al., 2022). The elevated levels of plasma t-tau in individuals with MCR suggested that MCR might share a similar pathology with AD.

For cognitively normal individuals, earlier studies have reported significant correlations between slow gait speed and amyloid deposition in the subcortical and cortical areas (Del Campo et al., 2016; Wennberg et al., 2017). However, recent studies did not find increased amyloid deposition in older adults with MCR (Bommarito et al., 2022; Gomez et al., 2022). In this study, for the first time, we compared plasma Aβ42 among older adults with NC, individuals with MCR and those with AD. It was found that, compared to the NC group, there were no significantly increased levels of plasma Aβ42 in the MCR group. We also noted that there were no significant correlations between the level of plasma Aβ42 and cognitive function in the MCR group. Previous studies suggested that the pathophysiological mechanisms underlying MCR were probably heterogeneous (Bommarito et al., 2022). Gomez et al. (2022) recruited 204 participants with MCR and noted that MCR was associated with prominent white matter abnormalities and frontoparietal atrophy. A cross-sectional study recruited 38 participants with MCR and reported that lacunar infarcts in the frontal lobe were associated with MCR in older adults (Wang et al., 2016). In the present exploratory study, the absence of an increase in the plasma Aβ42 in older adults with MCR suggested that MCR was probably not due to a pure AD pathology.

In this study, we further reported that higher levels of plasma t-tau were correlated with lower cognitive function in the MCR group. Mattsson et al. (2016) enrolled 1,284 participants, including healthy older adults, participants with cognitive impairment, and individuals with AD, in an exploratory study. They found that MMSE scores were negatively correlated with plasma t-tau concentrations. Another cross-sectional study using IMR assays to measure the plasma t-tau levels in participants with AD found that t-tau levels and MMSE scores had a strong negative correlation (Jiao et al., 2020). Chen et al. (2019) recruited 22 participants with amnestic MCI in a cohort study. They found that higher levels of t-tau were correlated with lower cognitive performance at the baseline and greater cognitive decline in 1.5 years follow-up. Tsai et al. (2019) enrolled 13 older adults with NC, 40 participants with amnestic MCI, and 37 individuals with AD, in a cross-sectional study. They found that MMSE scores were negatively correlated with plasma levels of t-tau. A retrospective case study noted that the MoCA scores in the lowest CSF tau quartile group were significantly higher than those in the highest quartile group (Pillai et al., 2019). Our results were in line with the findings of these studies, suggesting that cognitive performance might be correlated with the level of t-tau.

Prior studies have indicated that depression is not only a risk factor for AD (Gallagher et al., 2018), but also a symptom of AD (Van der Mussele et al., 2013). Many studies have investigated the relationship between depression and AD biomarkers. A systematic review including 15 studies reported significant differences in the Aβ levels between depressed and non-depressed older adults (Harrington et al., 2015). Babulal et al. (2020) enrolled 301 cognitively normal older adults in a cross-sectional study. They found that tau was associated with a depression diagnosis. In the present study, we observed a significant relationship between plasma t-tau and depression in the NC group. These findings were in line with the results from the previous studies.

There are some study limitations that should be addressed in this study. The first limitation was small sample size, which limits the statistical power. Studies with a larger sample size should be conducted in the future to confirm our results. A second limitation of this study is the cross-sectional study design, which prevented us from investigating the changes in plasma biomarkers and cognitive function in the MCR group over time. A large cohort study is warranted and encouraged. Third, the current study did not provide neuroimaging data. Therefore, we were unable to test the relationship between plasma biomarkers and brain pathology.

In conclusion, we found significantly higher plasma t-tau proteins in the MCR and AD groups, compared to the NC group, and correlations between the levels of plasma t-tau and cognitive function in individuals with MCR. These results implied that MCR and AD may share similar pathology. However, these findings need further confirmation in longitudinal studies.

## Data availability statement

The raw data supporting the conclusions of this article will be made available by the authors, without undue reservation.

## Ethics statement

The studies involving human participants were reviewed and approved by Institutional Review Board of MacKay Memorial Hospital. The patients/participants provided their written informed consent to participate in this study.

## Author contributions

P-HC and F-YC conceived and designed the experiments and wrote the manuscript. P-HC, Y-YL, and W-LH performed the experiments. F-YC and S-IL analyzed the data. All authors reviewed the manuscript and approved the submitted version.
