# Corrigendum: Associations between blood-based biomarkers of Alzheimer's disease with cognition in motoric cognitive risk syndrome: A pilot study using plasma Aβ42 and total tau

**DOI:** 10.3389/fnagi.2022.1125201

**Published:** 2023-01-05

**Authors:** Pei-Hao Chen, Sang-I Lin, Ying-Yi Liao, Wei-Ling Hsu, Fang-Yu Cheng

**Affiliations:** ^1^Department of Neurology, MacKay Memorial Hospital, Taipei, Taiwan; ^2^Department of Medicine, MacKay Medical College, New Taipei City, Taiwan; ^3^Graduate Institute of Mechanical and Electrical Engineering, National Taipei University of Technology, Taipei, Taiwan; ^4^Institute of Long-Term Care, MacKay Medical College, New Taipei City, Taiwan; ^5^Department of Gerontological Health Care, National Taipei University of Nursing and Health Sciences, Taipei, Taiwan; ^6^Center of Dementia Care, MacKay Memorial Hospital, New Taipei City, Taiwan

**Keywords:** plasma biomarker, cognition, gait speed, Alzheimer's disease, motoric cognitive risk syndrome

In the published article, an author's name was incorrectly written as Sang-Yi Lin. The correct spelling is Sang-I Lin.

The authors apologize for this error and state that this does not change the scientific conclusions of the article in any way. The original article has been updated.

